# Comparative Evaluation of Bivalent Malaria Rapid Diagnostic Tests versus Traditional Methods in Field with Special Reference to Heat Stability Testing in Central India

**DOI:** 10.1371/journal.pone.0058080

**Published:** 2013-03-05

**Authors:** Neeru Singh, Praveen K. Bharti, Mrigendra P. Singh, Sweta Mishra, Man M. Shukla, Ravendra K. Sharma, Rajesh K. Singh

**Affiliations:** 1 Regional Medical Research Centre for Tribals, Nagpur Road, Garha, Jabalpur, Madhya Pradesh, India; 2 National Institute of Malaria Research Field Unit Jabalpur, RMRCT Campus, Nagpur Road, Garha, Jabalpur, Madhya Pradesh, India; 3 Madhya Pradesh Technical Assistance Support Team (TAST), A/5, BDA Colony, Tulsi Nagar, Bhopal, Madhya Pradesh, India; Tulane University School of Public Health and Tropical Medicine, United States of America

## Abstract

**Background:**

Malaria presents a diagnostic challenge in areas where both *Plasmodium falciparum* and *P.vivax* are co-endemic. Bivalent Rapid Diagnostic tests (RDTs) showed promise as diagnostic tools for *P.falciparum* and *P.vivax*. To assist national malaria control programme in the selection of RDTs, commercially available seven malaria RDTs were evaluated in terms of their performance with special reference to heat stability.

**Methodology/Principal Findings:**

This study was undertaken in four forested districts of central India (July, 2011– March, 2012). All RDTs were tested simultaneously in field along with microscopy as gold standard. These RDTs were stored in their original packing at 25°C before transport to the field or they were stored at 35°C and 45°C upto 100 days for testing the performance of RDTs at high temperature. In all 2841 patients with fever were screened for malaria of which 26% were positive for *P.falciparum*, and 17% for *P.vivax*. The highest sensitivity of any RDT for *P.falciparum* was 98% (95% CI; 95.9–98.8) and lowest sensitivity was 76% (95% CI; 71.7–79.6). For *P.vivax* highest and lowest sensitivity for any RDT was 80% (95% CI; 94.9 - 83.9) and 20% (95% CI; 15.6–24.5) respectively. Heat stability experiments showed that most RDTs for *P.falciparum* showed high sensitivity at 45°C upto 90 days. While for *P.vivax* only two RDTs maintained good sensitivity upto day 90 when compared with RDTs kept at room temperature. Agreement between observers was excellent for positive and negative readings for both *P.falciparum* and *P.vivax* (Kappa >0.6–0.9).

**Conclusion:**

This is first field evaluation of RDTs regarding their temperature stability. Although RDTs are useful as diagnostic tool for *P.falciparum* and *P.vivax* even at high temperature, the quality of RDTs should be regulated and monitored more closely.

## Introduction

Malaria due to both *Plasmodium falciparum* and *P. vivax* is a life threatening disease for individual with low immunity [1], [2]. However, it is usually curable if diagnosed quickly [3], [4], [5]. The importance of obtaining results quickly from the examination of blood samples from suspected malaria patients is now made possible with the introduction of Rapid Diagnostic Tests (RDTs). Malaria RDTs were introduced in the nineties and have undergone many improvements [6], [7], [8]. The number of RDTs available, and the scale of their use has rapidly increased over the past few years [9], [10]. By now more than 60 RDT brands and over 200 different products have been developed [11] and the number of malaria RDTs produced annually has increased from 45 million in 2008 to 88 million in 2010 [12]. RDTs are hand held cassettes detecting Plasmodium parasites by an antibody antigen reaction [13]. These RDTs are available in several formats (lateral flow cassette, dipstick & cards etc) detecting one or more antigens (HRP-2 or pLDH or Aldolase or in combination). Although RDTs showed promise as new diagnostic tools, it is not clear which RDT is most appropriate for different epidemiological settings where both *P. vivax* and *P. falciparum* are co-endemic. Presumptive treatment of all fevers as malaria with chloroquine (CQ) becomes increasingly popular in resource poor setting because of lack of laboratory infrastructure and technical expertise [14], [15]. In such settings, empirical treatment results in substantial overuse of anti-malarial drugs and delays the diagnosis of other febrile illness [16], [17].

Malaria RDTs have the potential to provide a huge step forward in the management of febrile illness in malaria-endemic areas [12]. However, declining sensitivity of RDTs from field was also reported [18], [19]. There are several possible reasons for this i.e. HRP-2 gene deletions [20], [21] exposure to high temperature with or with out high humidity [22], operational difficulties and human error. High humidity accelerates denaturation [12], [23]. Therefore, to assist national malaria control programmes and other procurement agencies in the selection of products appropriate to their needs, commercially available bivalent malaria RDTs were evaluated in terms of their performance with special reference to stability testing in forest villages of Central India (Madhya Pradesh).

## Materials and Methods

**Figure 1:**
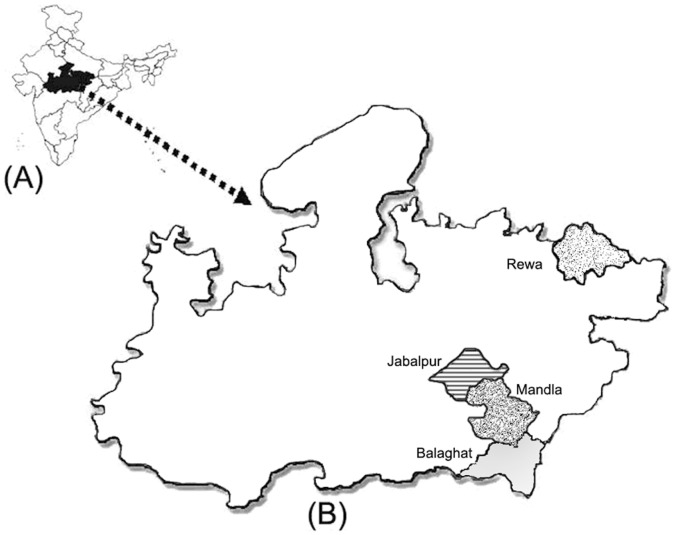
Map of India (A) Map of Madhya Pradesh (B) showing study areas.

This study was carried out in tribal and forested areas of four malarious districts of Madhya Pradesh i.e. Jabalpur, Mandla, Balaghat and Rewa during July 2011 to March 2012 (Figure 1). A cross sectional study design was used for assessing the performance of RDTs. Bivalent commercially available RDTs were selected for this study. The seven RDTs used in this study are – FIRST RESPONSE® malaria antigen pLDH/HRP2 combo card test (Premier medical corporation Ltd, Daman), GENOMIX(Pf/Pv) Malaria Antigen Detection test cassette (GENOMIX Molecular Diagnostics Pvt. Ltd., Hyderabad, Andhra Pradesh. FalciVax Rapid test for malaria Pv/Pf Device (Zephyr Biomedical, Verna, Goa), Parascreen®, Rapid test for malaria Pan/Pf Device (Zephyr Biomedicals, Verna, Goa), ParaHIT® Total Pan/Pf (Span Diagnostics Ltd., Surat, Gujarat), SD BIOLINE malaria Ag Pf/Pan (SD Bio Standard Diagnostics Pvt Ltd., Gurgaon, Haryana) and NecVIPARUM one step malaria Pf/Pv antigen detection test (Nectar Life Science Ltd., Chandigarh). These RDTs were procured directly from the manufacturers or their authorized dealers and stored in their original packing at 25°C before transport to the field sites (Table S1).

A one week training workshop was organized in the month of July 2011 to train the project staff for standard procedure of data collection and result interpretation. This was followed by two days training in field for screening and enrollment of study subjects, taking consent, collection of blood samples and RDT test procedures. The field clinic traveled 10 villages of each district for screening patients. All households of selected villages were visited by survey team and all clinically suspected malaria cases (as per National Programme guideline) were enrolled for the study. There were two teams, each consisting of two field workers, one technician and one research assistant.

All 7 RDTs were tested simultaneously in field on all enrolled patients using whole blood collected by a finger prick in a single bleed after taking informed consent. RDTs were performed at each site by members of the study team i.e. field worker and technician as per manufacturers instruction, and results interpreted and recorded after 15 to 30 minutes. They were advised that if the background of the RDT test window remained pink after 15 min, they should wait till the background is clear before declaring the test results. The study team recorded each RDT result as either positive or negative or invalid. Thick and thin smears were prepared from the same finger prick blood and air-dried. At the central laboratory of Regional Medical Research Centre for Tribals, thick and thin smears were stained with JSB [24] and examined by microscopist who was unaware of the RDT results. The microscopist examined 100 microscopic field of thick smear before declaring a smear as negative. When results of the RDT and microscopy were discrepant, smears were reviewed by a second independent microscopist unaware of previous result. Parasite densities were calculated according to the standard method (parasite/µl = no. of asexual parasites × 8000/no. of WBC counted). All positive and 10% negative blood smears and all the discordant results on presence or absence of parasitaemia between the two microscopists were resolved by referring to a third expert microscopist. These microscopists, examining blood smears were blinded to result of RDT, clinical status of patients and the microscopy.

### Ethics Statement

This study was approved by institutional review board of Regional Medical Research Centre for Tribals, Jabalpur India (IRB00006471). Written informed consent were obtained from all participants or the parents of children younger than 18 years, and assent were taken from children of age between 7 to <18 years as per Ethical Guidelines of Indian Council of Medical Research, New Delhi India.

### Heat Stability Testing

For temperature stability testing of the RDTs which were stored at 25°C on receipt, were then allocated to separate groups for storage at 35°C & 45°C upto100 days and at 60°C for 48 hrs. The incubators were stabilized at the required temperature for three days before the RDTs were placed inside. The log book was maintained for these incubators and temperature was monitored 3 times in a day. RDTs were removed from storage at 15 to 30 days time intervals allowed to reach at room temperature before testing. For all temperature/time combinations studied seven RDTs were tested for each temperature/time point and comparison was made with control RDTs (one for each temperature/time point). A micropipette was used to measure blood volume. The reading of the tests was undertaken by the same person to minimize inter-operator variability. The temperature during transport and in field were not monitored as it was not controlled.

### Sample Size

We assumed that the sensitivity of RDT is approximately 90% (P = 0.90) and absolute precision of 5% (d = 0.05), i.e. the estimated sensitivity of RDT will vary between 85–95%. The minimum required number of cases (positive cases by gold standard) is 138. Our past experiences in the study area revealed that *P. falciparum* is more prevalent than *P. vivax* in most parts of Madhya Pradesh. Thus, assuming slide falciparum rate (SFR) as 25% and slide vivax rate (SVR) as 15% for the study area, we need to screen about 552 cases for *P. falciparum* and 920 cases for *P. vivax*. Thus, overall we need to screen about 1500 malaria suspected cases to test the sensitivity of different RDTs for both *P. falciparum* and *P. vivax*.

For heat stability testing, we assumed that the sensitivity of RDT kit is approximately 90% (P = 0.90) and absolute precision of 10% (d = 0.1), i.e. the estimated sensitivity of RDT kit will vary between 80–100%. The minimum required number of cases (positive cases by gold standard) is 35 at each level of temperature.

### Treatment

All patients infected with *P. falciparum* and *P. vivax* were given treatment as per treatment guideline of National Vector Borne Disease Control Programme (NVBDCP) [25]. All adult subjects with *P. falciparum* were administrated the oral dose of ACT (1500 mg Sulfadoxine, 75 mg Pyrimethamine and 600 mg of Artesunate divided into 3 days) with single dose of Primaquine (45 mg). *P. vivax* cases were given 1,500 mg Chloroquine for three days, followed by 15 mg Primaquine daily for 14 days. Infants and children were given proportionally lower doses. Infants and pregnant women were not given Primaquine.

### Data Entry and Analysis

The forms were double-entered using CS-Pro 4.1 (US Census Bureau, Washington, DC, USA), with range, consistency, and edit checks built into the data entry programme for quality control. The two databases were validated and all inconsistencies and differences were resolved. Statistical analyses were performed using STATA 11.2 (StataCorp Texas USA). Diagnostic performance characteristics i.e. sensitivity, specificity and positive & negative predictive values (PPV & NPV) were calculated against light microscopy as gold standard by using ‘diagt’ command in STATA. However for heat stability testing sensitivity and specificity were calculated against diagnostic performance of RDTs kept at room temperature.

During calculation of sensitivity/specificity, matching mixed infections with *P. falciparum* and *P. vivax* were taken as true positive for both *P. falciparum* and *P. vivax* species and *P. falciparum* only gametocyte cases were considered as true negative. *P. malariae* infections were excluded during the analysis of RDTs performance. Inter-observer agreement for both results of positive and negative reading as well as for stability was expressed by Kappa statistics for each pair of observers. A Kappa between 0.6 and 0.8 was considered a good agreement, higher than 0.8 was considered as excellent [26].

## Results

In all 2841 patients aged 2 month to 80 years (median age 10 years) with fever/history of fever were screened for malaria. Only 2207 eligible subjects were enrolled for RDT testing in parallel with microscopy. The enrollment of study subjects are divided into two parts i.e. one for testing RDT diagnostic performance under normal field condition and another for heat stability testing by keeping RDTs at various temperature up to 100 days.

### Study Part I

In this part of the study, 1807 subjects were tested by all 7 RDTs in field. Of which 46.1% were positive, 25.7% *P. falciparum*, 16.6% *P. vivax,* 1.0% *P. malariae*, 1.9% mixed infection of *P. falciparum* and *P. vivax* and 0.9% mixed infection of *P. malaria*e with *P. falciparum* and/or *P. vivax*. FIRST RESPONSE® detected 468 out of 480 microscopically confirmed asexual±sexual falciparum malaria infection (Table 1). However, other RDTs i.e. FalciVax detected 429, parascreen® 425, SD BIOLINE & NecVIPARUM 416, ParaHIT® Total 373 and GENOMIX detected only 360 falciparum infections (Table 1). Among microscopically confirmed 329 *P. vivax* subjects, FIRST RESPONSE® detected 262, parascreen® 162, SD BIOLINE 163, FalciVax 149, NecVIPARUM 141, ParaHIT® Total 112, while only 65 cases were detected by GENOMIX (Table 2). Number of invalid test (absence of control band) was recorded in 1, 7, 2, 8, 9, 15 and 4 tests respectively for FIRST RESPONSE®, parascreen®, ParaHIT® Total, FalciVax, SD BIOLINE, GENOMIX and NecVIPARUM.

**Table 1:** Evaluation of *P. falciparum* by seven different RDTs against light microscopy as gold standard in four district of Madhya Pradesh, central India.

	FIRST RESPONSE®	Parascreen®	ParaHIT® Total	FalciVax	SD BIOLINE	GENOMIX	NecVIPARUM
Sensitivity (95% CI)	97.7 (95.9–98.8)	88.9 (85.7–91.6)	77.9 (73.9–81.5)	89.4 (86.3–92.0)	86.8 (83.5–89.7)	75.8 (71.7–79.6)	86.7 (83.3–89.6)
Specificity (95% CI)	90.1 (88.4–91.7)	85.8 (83.8–87.6)	90.6 (88.9–92.1)	84.1 (82.0–86.0)	85.4 (83.3–87.2)	91.9 (90.3–93.3)	89.6 (87.9–91.2)
PPV (95% CI)	78.1 (74.6–81.4)	69.3 (65.5–73.0)	74.9 (70.8–78.7)	67.1 (63.3–70.8)	68.3 (64.4–72.0)	77.1 (73.0–80.8)	75.2 (71.4–78.8)
NPV (95% CI)	99.1 (98.4–99.5)	95.5 (94.2–96.6)	91.9 (90.3–93.3)	95.6 (94.3–96.7)	94.7 (93.3–95.9)	91.3 (89.7–92.8)	94.9 (93.5–96.0)
TP	468	425	373	429	416	360	416
TN	1196	1134	1201	1109	1126	1210	1186
FP	131	188	125	210	193	107	137
FN	11	53	106	51	63	115	64

**Table 2:** Evaluation of *P. vivax* by seven different RDTs against light microscopy as gold standard in four district of Madhya Pradesh, central India.

	FIRST RESPONSE®	Parascreen®	ParaHIT® Total	FalciVax	SD BIOLINE	GENOMIX	NecVIPARUM
Sensitivity (95% CI)	79.6 (74.9–83.9)	49.5 (44.0–55.1)	34.3 (29.1–39.7)	45.7 (40.2–51.3)	49.5 (44.0–55.1)	19.8 (15.6–24.5)	42.9 (37.4–48.4)
Specificity (95% CI)	98.4 (97.7–99.0)	97.7 (96.8–98.4)	97.9 (97.0–98.6)	98.6 (97.9–99.2)	98.1 (97.3–98.7)	96.7 (95.6–97.5)	98.4 (97.6–99.0)
PPV (95% CI)	91.9 (88.1–94.8)	82.7 (76.6–87.7)	78.3 (70.7–84.8)	88.2 (82.3–92.6)	85.3 (79.5–90.0)	57.0 (47.4–66.3)	85.5 (79.1–90.5)
NPV (95% CI)	95.6 (94.4–96.6)	89.7 (88.1–91.2)	87.1 (85.4–88.6)	89.1 (87.5–90.6)	89.7 (88.1–91.1)	84.3 (82.5–86.0)	88.5 (86.9–90.0)
TP	262	162	112	149	163	65	141
TN	1454	1439	1447	1453	1441	1415	1450
FP	23	34	31	20	28	49	24
FN	67	165	215	177	166	263	188

The analysis of results revealed that the sensitivity of the FIRST RESPONSE® for *P. falciparum* was 98% (Table 1), of parascreen® and FalciVax 89%, SD BIOLINE & NecVIPARUM 87% and ParaHIT® Total 78%, whereas the sensitivity of GENOMIX was only 76%. The specificity for *P. falciparum* was 92% by GENOMIX, 91% by ParaHIT® Total, 90% by FIRST RESPONSE® and NecVIPARUM, 86% by parascreen®, 85% by SD BIOLINE and 84% by FalciVax.

For *P. vivax*, the sensitivity of different tests when compared with microscopy were 80% by FIRST RESPONSE®, 50% by parascreen® and SD BIOLINE, 46% by FalciVax, 43% by NecVIPARUM and 34% by ParaHIT® Total, while only 20% by GENOMIX (Table 2). Specificity of all these tests ranged between 97–99%.

**Figure 2:**
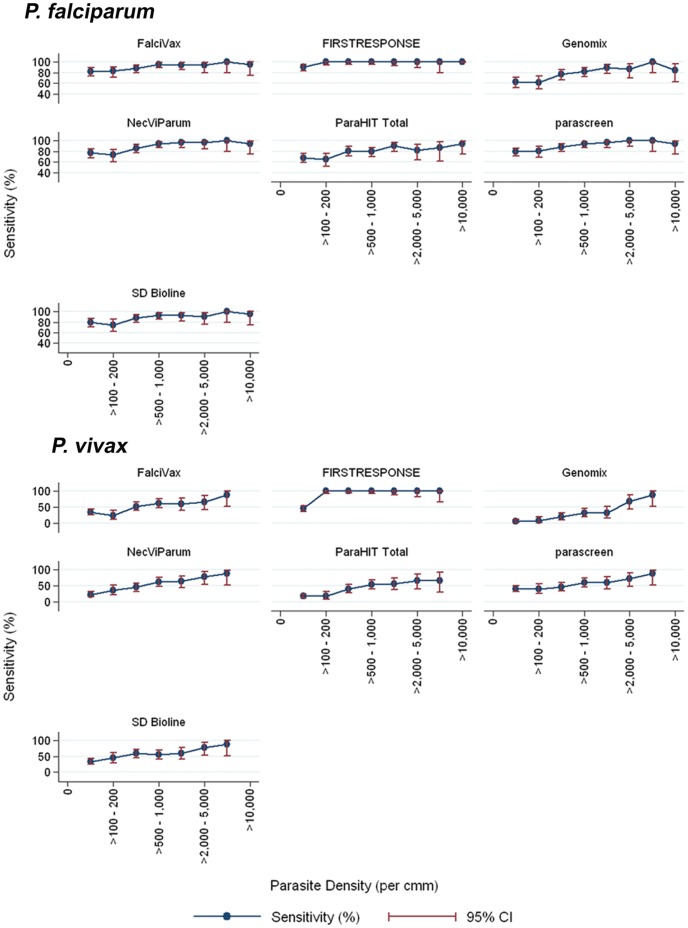
Parasite density and Sensitivity of seven RDTs of *P. falciparum* and *P. vivax*.

Analysis of sensitivity on different level of parasitaemia revealed that FIRST RESPONSE® was able to detect 100% malaria infection at >100 parasites/µl of blood for both *P. falciparum* and *P. vivax*. While parascreen®, FalciVax, SD BIOLINE and NecVIPARUM were able to detect >90% *P. falciparum* infections when parasite densities were >500 parasites/µl. However, ParaHIT® Total and GENOMIX detects 90% *P. falciparum* infections when parasitaemia was >1000 parasites/µl. Regarding *P. vivax* infections except FIRST RESPONSE®, other RDTs detect only 31–63% when parasite density is >500 parasites/µl (Figure 2).

### Study Part II

RDTs kept at 35°C and 45°C for 15, 30, 60, 90 and 100 days and at 60°C for 48 hours for heat stability test in the field. Seventy five clinically suspected malaria cases were tested on 15, 30, 60 and 90 days, and 50 clinically suspected cases were tested on 100 days and at 60°C for 48 hours intervals.

Results of heat stability testing was shown in Table 3 & 4. Experiments showed that sensitivity of most of the RDTs for *P. falciparum* was very good (>90) both at 35° and 45°C up to day 90 when compared with RDTs kept at room temperature. However, a sharp decline in sensitivity was recorded on day 100 at 35°C and 45°C by most of the RDTs. On the contrary all RDTs kept at 60°C for 48 hours showed no decline in the diagnostic performance.

**Table 3:** Sensitivity of 7 different RDTs at 35°C, 45°C and 60°C for *P. falciparum* against Room Temperature.

Time Intervals	Temp	FIRST RESPONSE®		Parascreen®		ParaHIT® Total		FalciVax		SD BIOLINE		GENOMIX		NecVIPARUM	
		Sens.	Spec.	Sens.	Spec.	Sens.	Spec.	Sens.	Spec.	Sens.	Spec.	Sens.	Spec.	Sens.	Spec.
		(95%CI)	(95%CI)	(95%CI)	(95%CI)	(95%CI)	(95%CI)	(95%CI)	(95%CI)	(95%CI)	(95%CI)	(95%CI)	(95%CI)	(95%CI)	(95%CI)
**15 days**	**35°C**	93	100	89	90	72	91	84	87	81	94	100	100	96	100
		(77–99)	(92–100)	(73–97)	(76–97)	(53–86)	(78–97)	(69–94)	(71–96)	(65–91)	(80–99)	(86–100)	(93–100)	(81–100)	(93–100)
	**45°C**	93	97	85	93	75	93	82	87	78	94	100	100	93	98
		(77–99)	(89–100)	(69–95)	(80–98)	(57–89)	(81–99)	(66–92)	(71–96)	(62–89)	(80–99)	(86–100)	(93–100)	(76–99)	(89–100)
**30 days**	**35°C**	95	100	98	94	94	98	95	100	92	100	89	95	94	97
		(83–99)	(90–100)	(87–100)	(80–99)	(80–99)	(87–100)	(84–99)	(89–100)	(78–98)	(91–100)	(74–97)	(82–99)	(81–99)	(87–100)
	**45°C**	97	100	95	97	88	98	93	100	87	95	89	97	89	97
		(87–100)	(90–100)	(84–99)	(84–100)	(73–97)	(87–100)	(81–99)	(89–100)	(71–96)	(82–99)	(74–97)	(86–100)	(74–97)	(87–100)
**60 days**	**35°C**	91	97	93	100	81	92	93	100	77	94	80	91	78	97
		(77–97)	(84–100)	(82–99)	(88–100)	(65–92)	(79–98)	(82–99)	(88–100)	(62–89)	(79–99)	(64–91)	(77–98)	(62–89)	(85–100)
	**45°C**	93	94	93	100	75	90	93	100	74	94	69	91	73	91
		(81–99)	(79–99)	(81–99)	(88–100)	(58–88)	(75–97)	(82–99)	(88–100)	(59–87)	(79–99)	(52–83)	(77–98)	(57–86)	(76–98)
**90 days**	**35°C**	96	100	98	92	88	92	98	92	92	100	95	90	98	100
		(86–100)	(87–100)	(89–100)	(75–99)	(75–95)	(75–99)	(89–100)	(74–99)	(80–98)	(84–100)	(84–99)	(74–98)	(89–100)	(88–100)
	**45°C**	94	96	96	92	64	96	94	100	90	86	90	90	98	96
		(83–99)	(81–100)	(86–100)	(75–99)	(49–77)	(80–100)	(84–99)	(86–100)	(78–97)	(65–97)	(76–97)	(73–98)	(89–100)	(82–100)
**100 days**	**35°C**	88	100	88	92	60	88	84	92	80	100	82	100	82	89
		(69–98)	(86–100)	(69–98)	(74–99)	(39–79)	(69–98)	(64–96)	(74–99)	(59–93)	(86–100)	(60–95)	(88–100)	(60–95)	(72–98)
	**45°C**	76	100	84	96	56	92	80	96	84	100	64	100	82	100
		(55–91)	(86–100)	(64–96)	(80–100)	(35–76)	(74–99)	(59–93)	(80–100)	(64–96)	(86–100)	(41–83)	(88–100)	(60–95)	(88–100)
**48 hr**	**60°C**	92	93	97	93	86	93	95	85	95	92	90	90	94	75
		(78–98)	(66–100)	(86–100)	(66–100)	(71–95)	(66–100)	(82–99)	(55–98)	(82–99)	(64–100)	(74–98)	(67–99)	(80–99)	(48–93)

**Table 4:** Sensitivity of 7 different RDTs at 35°C, 45°C and 60°C for *P. vivax* against Room Temperature.

Time Intervals	Temp	FIRST RESPONSE®		Parascreen®		ParaHIT® Total		FalciVax		SD BIOLINE		GENOMIX		NecVIPARUM	
		Sens.	Spec.	Sens.	Spec.	Sens.	Spec.	Sens.	Spec.	Sens.	Spec.	Sens.	Spec.	Sens.	Spec.
		(95%CI)	(95%CI)	(95%CI)	(95%CI)	(95%CI)	(95%CI)	(95%CI)	(95%CI)	(95%CI)	(95%CI)	(95%CI)	(95%CI)	(95%CI)	(95%CI)
**15 days**	**35°C**	100	100	83	94	71	93	71	91	73	93	91	98	84	95
		(86–100)	(93–100)	(61–95)	(84–99)	(42–92)	(84–98)	(48–89)	(80–97)	(45–92)	(84–98)	(59–100)	(92–100)	(60–97)	(85–99)
	**45°C**	96	98	87	94	71	95	71	91	73	97	91	98	79	95
		(80–100)	(89–100)	(66–97)	(84–99)	(42–92)	(86–99)	(48–89)	(80–97)	(45–92)	(89–100)	(59–100)	(92–100)	(54–94)	(85–99)
**30 days**	**35°C**	97	100	87	98	71	97	73	93	79	95	75	97	68	89
		(86–100)	(91–100)	(60–98)	(91–100)	(44–90)	(88–100)	(45–92)	(84–98)	(54–94)	(85–99)	(48–93)	(88–100)	(43–87)	(78–96)
	**45°C**	94	97	87	98	59	91	73	97	84	98	75	95	74	89
		(81–99)	(86–100)	(60–98)	(91–100)	(33–82)	(81–97)	(45–92)	(89–100)	(60–97)	(90–100)	(48–93)	(86–99)	(49–91)	(78–96)
**60 days**	**35°C**	100	98	90	99	82	97	89	100	89	99	63	91	88	97
		(86–100)	(89–100)	(56–100)	(92–100)	(48–98)	(89–100)	(52–100)	(95–100)	(52–100)	(92–100)	(25–92)	(81–97)	(47–100)	(90–100)
	**45°C**	100	100	90	100	64	94	89	100	89	99	75	96	88	99
		(86–100)	(93–100)	(56–100)	(94–100)	(31–89)	(85–98)	(52–100)	(95–100)	(52–100)	(92–100)	(35–97)	(87–99)	(47–100)	(92–100)
**90 days**	**35°C**	100	100	88	100	44	94	60	99	50	98	36	100	64	100
		(87–100)	(93–100)	(47–100)	(95–100)	(14–79)	(85–98)	(26–88)	(92–100)	(16–84)	(91–100)	(13–65)	(94–100)	(31–89)	(94–100)
	**45°C**	100	100	75	100	33	97	40	95	50	98	36	100	64	100
		(87–100)	(93–100)	(35–97)	(95–100)	(8–70)	(90–100)	(12–74)	(87–99)	(16–84)	(92–100)	(13–65)	(94–100)	(31–89)	(94–100)
**100 days**	**35°C**	92	95	60	96	25	96	33	96	57	98	67	93	67	98
		(62–100)	(82–99)	(15–95)	(85–100)	(1–81)	(85–100)	(1–91)	(86–100)	(18–90)	(88–100)	(22–96)	(81–99)	(9–99)	(89–100)
	**45°C**	92	97	40	93	25	96	33	94	57	98	33	96	33	96
		(62–100)	(86–100)	(5–85)	(82–99)	(1–81)	(85–100)	(1–91)	(83–99)	(18–90)	(88–100)	(4–78)	(85–99)	(1–91)	(86–100)
**48 hr**	**60°C**	100	100	100	100	33	98	50	96	67	98	80	96	50	98
		(63–100)	(92–100)	(29–100)	(93–100)	(1–91)	(89–100)	(8–99)	(86–100)	(9–99)	(89–100)	(28–100)	(85–100)	(1–99)	(89–100)

**Figure 3:**
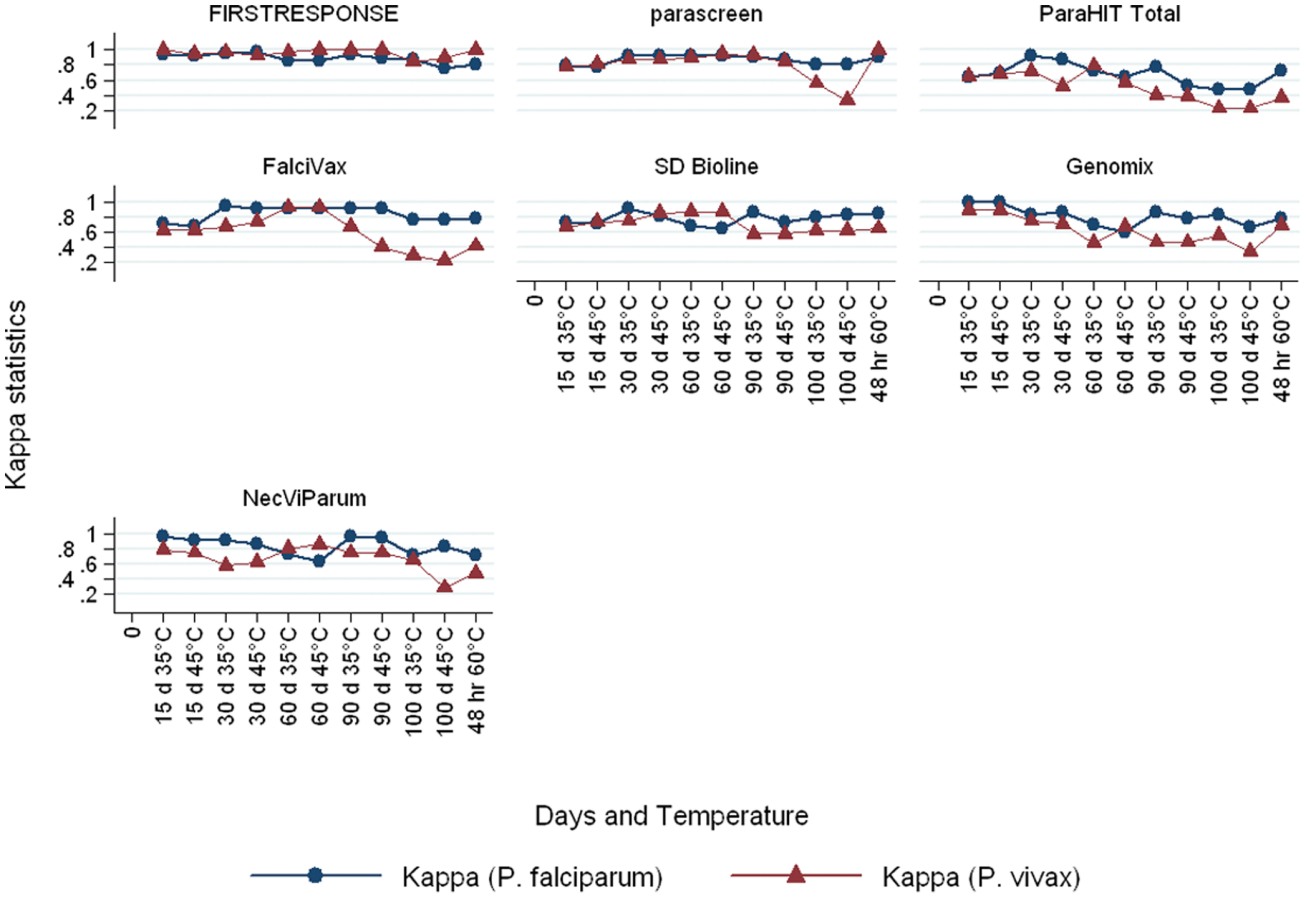
Kappa statistics of measure of agreement at temperature 35°C, 45°C and 60°C against room temperature for *P. falciparum* and *P. vivax* of seven difference RDTs.

For *P. vivax*, the sensitivity of FIRST RESPONSE® was very good up to 90 days (100%) and a decline was noticed on day 100 (92%). Parascreen also performed well upto day 90 at 35°C (88%). However, a sharp decline in sensitivity was observed on day 90 at 45°C (75%). While other RDTs showed a steady decline in sensitivity from day 60 onwards (Table 4). The overall agreement and Kappa values between pairs of observers were very good for both at 35°and 45°C for *P. falciparum*. However, Kappa values was not good for some RDTs for *P. vivax* especially on days 90 and 100 (Figure 3).

## Discussion

A number of studies on RDTs have been conducted, although measures of accuracy have varied widely, as a result of differences in methodology, study site epidemiology and type of RDT used i.e. histidine rich protein - 2 (HRP-2) and plasmodium specific lactate dehydrogenase (pLDH) and species specific pLDH or aldolase based test [27], [28], [17]. Ideally, bivalent RDTs will help to target anti-malarial treatment for *P. vivax* and *P. falciparum* by grass root workers who provide on the spot diagnosis and treatment in areas where microscopy cannot be established. The required sensitivity of a test may also vary with species, a less sensitive test may be acceptable for detection of *P. vivax* compared to detection of *P. falciparum* as severe outcomes due to missed diagnosis are less likely [29].

The present study assessed the performance of various bivalent RDTs in field population. Overall, highest sensitivity for *P. falciparum* was >95% and for *P. vivax* ≥80%. The high frequency of positive smears in this study is consistent with previous studies in Central India [30]. Likewise the sensitivity and specificity estimates are consistent with previous studies [10]. Further, when the results of this study was compared with WHO, FIND and CDC product testing of RDTs, where the evaluation was performed against a standardized panel of cultured *P. falciparum* and frozen blood samples (200–2000 parasites/µl of blood) by experienced technicians in a research laboratory and not in field as done in the present study, the results of the WHO, FIND and CDC are comparable to this study with regard to diagnostic performance of five RDTs evaluated in both the studies. Additionally, first field based evaluation of commercially available RDTs regarding their temperature stability was also carried out in the present study. Among the 7 bivalent RDTs, most RDTs for *P. falciparum* showed a very high sensitivity over period at 35 and 45°C upto day 90 in terms of percentage of successful tests. While for *P. vivax*, the FIRST RESPONSE maintained good sensitivity over period at 35, 45°C upto day 90 and showed a decline in sensitivity on day 100. Parascreen was also good upto day 90 at 35°C only and showed a sharp decline in sensitivity on day 100. These results are consistent with those of WHO, FIND and CDC product testing of RDTs.

Heat stability is vital to maintaining sensitivity of the test in the field [22], [30]. HRP-2 is a very stable antigen [31], while pLDH may degrade during long storage [11], [22]. Wide variations in stability between various RDTs were recorded in this study. The lacks of quality control of RDTs present a risk to patients through incorrect diagnosis and inappropriate anti-malaria treatment [32]. As a result for procurement, it is essential that careful consideration be given to stability results to ensure that RDTs works under extreme temperature. All the RDTs in this evaluation were packaged in individual envelopes that contain a desiccant. This allows the health worker to open the envelope of a test at the time of use in field limiting exposure to high humidity [12] as the field trial for stability testing was carried out during peak rainy season. The stability testing results presented here provide assessment of both, stability of the RDT and also the quality of its packaging [12]. However, there are some potential limitations in generalizing our results to predict the success of implementing RDTs at high temperature. Though temperature was held constant in this evaluation, humidity was not maintained. Temperature and humidity in field fluctuate with time of day and season and 100 days storage may not accurately predict long term stability under field conditions. Loss of parasite detections over this period indicates that chances of decline in sensitivity cannot be overruled [12]. It is worthwhile to mention here that field trial was carried out during July–March thus all 3 seasons i.e. monsoon, autumn and summer were covered. An additional limitation of this study was that highly trained individual performed all the testing in this evaluation. In field settings malaria RDTs will often be used by health workers with limited training and supervision. Temperature upto 45°C is likely in uncontrolled storage in tropical countries and temperature may further increase during transport [33]. The overall agreement and Kappa values between pairs of observers were excellent for both positive and negative results. Likewise, for heat stability testing for *P. falciparum* generally agreement and Kappa values were good for all RDTs however, for *P. vivax* the Kappa was not good for most RDTs on day 90 and 100. Thus the use of RDTs widely in the programme will require considerable regulation and quality control [34]. Further research is required to determine why some RDTs examined were more susceptible to heat stress in order to improve their temperature stability.

In view of this, while malaria RDTs can be applied in a number of settings, the greatest potential for impact on public health is in extension of access to accurate, parasite based diagnosis of malaria to regions and populations where good quality microscopy based testing is impractical to maintain, making possible the implementation of recent WHO/NVBDCP recommendation on parasite based diagnosis prior to anti-malarial therapy [25], [35]. However, if the RDTs are to replace microscopy in field for malaria diagnosis, they must be able to work with high level of reliability at high ambient temperature. Diagnostic testing by microscopy or RDT to a level of 200 parasites/µl will reliably detect nearly all clinically relevant infections in malaria endemic areas [10], [36]. However, as some countries move towards elimination, population immunity will be decreased and it will increasingly important to use diagnostic tests that detects low parasite densities [17], [37], [38]. Therefore, the ability to detect low parasite density infections reliably therefore, remains important as malaria elimination initiative is increasing in several countries.

Nevertheless, the challenges associated with establishing the routine use of RDTs in rural remote setting where microscopy could not be established should be tackled carefully as the distribution of RDTs and antimalarials must occur hand in hand to ensure effective case management of febrile disease. These results also suggest that the quality of RDTs should be regulated and monitored more closely. We also noticed a wide range in pricing for RDTs ranging from 0.70$ to 2$. However, high price of RDTs is not an assurance of good performance. Therefore, continued search and eventually introducing other alternative and highly sensitive low cost malaria diagnostic methods should also be explored which are capable of detecting low parasitaemia at high ambient temperature.

## Supporting Information

Table S1 Details of bivalent Rapid Diagnostic Tests.
(DOC)
