# Supplementary material for: Comparative Evaluation of Bivalent Malaria Rapid Diagnostic Tests versus Traditional Methods in Field with Special Reference to Heat Stability Testing in Central India
Source: PLoS One. 2013 Mar 5;8(3):e58080. doi: 10.1371/journal.pone.0058080 (PMC3589473; doi:10.1371/journal.pone.0058080)
Supplement: Table S1 — Details of bivalent Rapid Diagnostic Tests. (DOC) [file pone.0058080.s001.doc]

**Table S1** Details of bivalent Rapid Diagnostic Tests

| **S. No.** | **RDTs** | **Manufacturer** | **Target Antigen** | **Batch No.** | **Date of Manufacture** | **Date of Expiry** | **Date of Procurement** | **Procurement from** | **Cat No.** |
| --- | --- | --- | --- | --- | --- | --- | --- | --- | --- |
| **1** | FIRST RESPONSE® Malaria pLDH/HRP2 combo | Premier Medical Corporation Ltd., Daman, India | Pan pLDH/Pf HRP II | 69G1011 | 2012-JAN | 2013-SEP | 21/07/2011 | Manufacturer | 116FRC30 |
|  |  |  |  | 69I0510 | 2010-SEP | 2012-APR | 12/12/2011 |  |  |
| **2** | Genomix Malaria Pf/Pv | Genomix Molecular Diagnostics Pvt. Ltd., Hyderabad, Andhra Pradesh, India | Pf HRP II/Pv pLDH | LAB0111-M | 2011-JAN | 2012-SEP | 27/07/2011 | Supplier | GM24RS |
|  |  |  |  | GM1111 | 2011-NOV | 2013-FEB | 13/12/2011 |  |  |
| **3** | FalciVax Rapid Test for Malaria Pv/Pf | Zephyr Biomedicals, Verna, Goa, India | Pf HRP II/Pv pLDH | 81113 | 2011-MAY | 2013-APR | 20/07/2011 | Supplier | 50300025 |
|  |  |  |  | 81119 | 2011-NOV | 2013-OCT | 18/12/2011 |  |  |
| **4** | Parascreen® Device (Pan/Pf) | Zephyr Biomedicals, Verna, Goa, India | Pf HRP II/PAN pLDH | 101185 | 2011-APR | 2013-MAR | 20/07/2011 | Supplier | 50310025 |
|  |  |  |  | V531003Z | 2011-NOV | 2013-OCT | 18/12/2011 |  |  |
| **5** | ParaHIT® Total | Span Diagnostics Ltd., Surat, Gujarat, India | Pf HRP II/ PAN Aldolase | 4000006730 | 2011-JUN | 2012-DEC | 26/07/2011 | Supplier | Old:25988  New:551C201-10 |
|  |  |  |  | 4000006883 | 2011-JULY | 2013-JAN | 13/12/2011 |  |  |
| **6** | SD Malaria Antigen Pf/Pan | SD Biostandard Diagnostics Pvt. Ltd., Gurgaon, Haryana, India | Pf HRP II/PAN pLDH | 90050 | 2010-NOV | 2012-NOV | 19/07/2011 | Supplier | 05FK60I-40 |
|  |  |  |  | 90065 | 2011-OCT | 2013-OCT | 07/12/2011 |  |  |
| **7** | NecViparum Pf/Pv | Nectar Lifesciences Ltd., Chandigarh, India | Pf HRP II/ Pv pLDH | W5600501W | 2010-OCT | 2012-OCT | 19/07/2011 | Supplier | --- |
|  |  |  |  | W5610604W | 2011-JUN | 2013-JUN | 07/12/2011 |  |  |

--- Not mentioned on the kits.
